# Conducting clinical trials during the COVID-19 pandemic—a collaborative trial network response

**DOI:** 10.1186/s13063-021-05200-0

**Published:** 2021-04-14

**Authors:** Laura Robison, Yeoungjee Cho, Andrea K. Viecelli, David W. Johnson, Carmel M. Hawley, Andrea Valks, Peta-Anne Paul-Brent, Ruth Stastny, Julie Varghese, Charani Kiriwandeniya, Elaine M. Pascoe, Liza A. Vergara, Magid A. Fahim, Neil Boudville, Rathika Krishnasamy, Donna Reidlinger, on behalf of the Australasian Kidney Trials Network

**Affiliations:** 1 Australasian Kidney Trials Network, The University of Queensland, Level 5, Translational Research Institute, 37 Kent Street, Woolloongabba, QLD 4102 Australia; 2 The Department of Nephrology, Princess Alexandra Hospital, Brisbane, QLD Australia; 3 The Translational Research Institute, Brisbane, QLD Australia; 4 Metro North Hospital & Health Service, Brisbane, QLD Australia; 5 The Medical School, University of Western Australia, Perth, WA Australia; 6 Department of Nephrology, Sunshine Coast University Hospital, Birtinya, QLD Australia

## Abstract

The unprecedented demand placed on healthcare systems from the COVID-19 pandemic has forced a reassessment of clinical trial conduct and feasibility. Consequently, the Australasian Kidney Trials Network (AKTN), an established collaborative research group known for conducting investigator-initiated global clinical trials, had to efficiently respond and adapt to the changing landscape during COVID-19. Key priorities included ensuring patient and staff safety, trial integrity and network sustainability for the kidney care community. New resources have been developed to enable a structured review and contingency plan of trial activities during the pandemic and beyond.

## Introduction

The COVID-19 pandemic presents a threat to the health and wellbeing of everyone, particularly in those diagnosed with a chronic medical condition, such as chronic kidney disease (CKD). People with CKD are at heightened risk of developing severe complications from contracting COVID-19 due to a high burden of co-morbidities and a compromised immune system [1]. Moreover, patients requiring facility haemodialysis are at heightened risk of exposure to COVID-19 during hospital or dialysis unit outbreaks [2], while kidney transplant patients are at higher risk of acquiring infections by virtue of anti-rejection medications suppressing their immune system [3].

During the COVID-19 pandemic, the Australasian Kidney Trials Network (AKTN), an established collaborative research group conducting investigator-initiated clinical trials in people with kidney disease [4], responded to the threat with a considered and coordinated approach that prioritised the wellbeing of the kidney community and researchers while maintaining trial integrity. At the time of the outbreak, there was no disaster management policy in place and trial risk assessments had not accounted for the impact of a major pandemic. Here, we describe new strategies and resources that were designed to support AKTN trials in response to the COVID-19 pandemic.

## AKTN background

The AKTN research portfolio primarily focuses on conducting clinical trials evaluating outcomes derived from the shared priorities of patients, caregivers, clinicians, researchers and policy makers [5]. Since its inception in 2005, the AKTN has coordinated 18 predominantly phase III–IV multicentre investigator-initiated trials in collaboration with international and local research partners (3 national and 15 international trials to date). More recent protocols have addressed the imperative for pragmatic trial design by collaborating with the Australia and New Zealand Dialysis and Transplant (ANZDATA) registry to embed the research into routine clinical practice, reducing the number of trial-specific assessments and bridging the divide between clinical and research-related activities. These registry-based trials also leverage existing registry data points, thereby reducing burden on sites by avoiding duplicate data entry [6]. Championing a pragmatic trial design [7] with less strict inclusion criteria was executed to enhance feasibility, efficiency, generalisability and implementation of study outcomes.

**Fig. 1:**
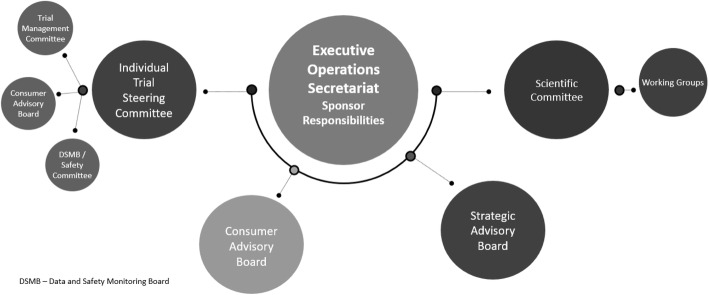
AKTN governance structure

A central coordinating office in Brisbane, Australia, houses the operational infrastructure and expertise necessary to coordinate multiple clinical trials in Australia, and internationally. Individual trials are overseen by a Trial Steering Committee comprising key clinical researchers whose decisions are informed by the day-to-day operational activities of a Trial Management Committee. The Trial Management Committee is responsible for ensuring that quality management, risk management and feasibility assessments are incorporated into AKTN trial design and governance procedures (Fig. 1).

Prior to the COVID-19 pandemic, site training was usually conducted face to face. Risk-based monitoring and quality by design [8] were applied to all AKTN trials and all trial monitoring plans pre-pandemic; this included at least one early on-site visit to conduct source data verification, establish compliance with Good Clinical Practice (GCP) and determine which areas of the risk assessment and monitoring plan may require adjustment. Depending on the risk level associated with the trial, the monitoring plans also incorporated further on-site visits and regular remote data monitoring to ensure trial integrity and patient safety.

## COVID-19 situation

COVID-19 emerged globally at a rapid rate, creating an unprecedented surge in patients presenting to healthcare services around the world. The deployment of clinical research staff to support the COVID-19 front line response and the strict physical distancing requirements necessitated that clinical trial operations be scaled back significantly. This posed questions regarding the viability of patient recruitment, study visits and assessments, patient safety monitoring, data collection and the long-term ongoing operations of the network due to the uncertainty of the health crisis duration.

## AKTN response

At the time of the COVID-19 outbreak, AKTN trials were at various stages of conduct: 5 were in development, 5 were recruiting and 6 in follow-up. The AKTN COVID-19 response required flexibility to accommodate the unique situation for each trial and the rapidly evolving situation. The priorities in the COVID-19 trial response management plans included first and foremost maintaining patient safety, staff health and wellbeing, trial integrity and business continuity.

### Initial response planning

**Fig. 2:**
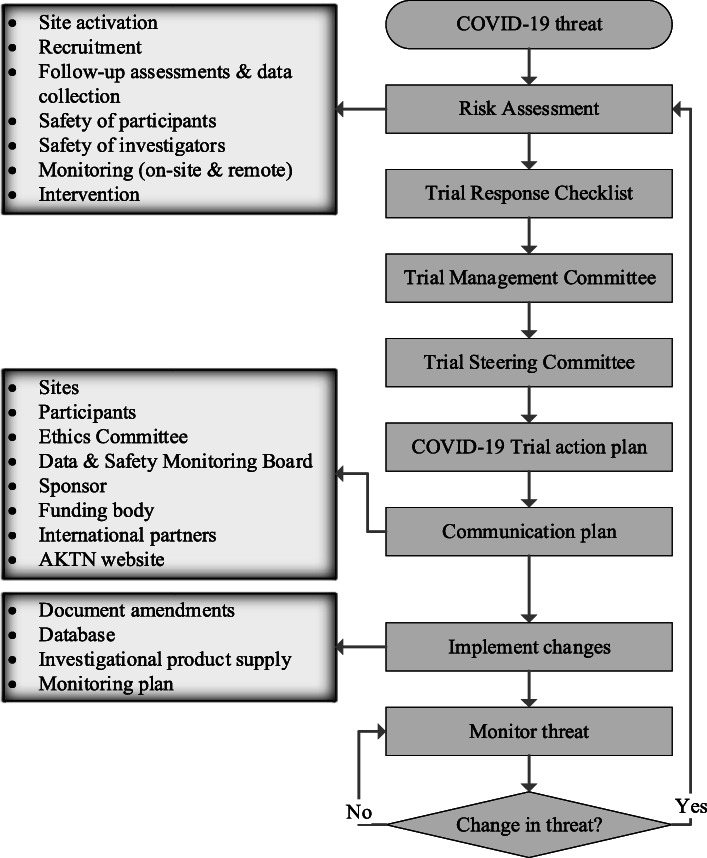
Summary of trial response process

Trial-specific COVID-19 action plans reflected the COVID-19 advice and information from regulatory bodies and ethics committees and upheld the regulations, guidelines [9–11], codes, policies and other standards applicable to clinical trials. Mindful that any key decisions and actions would need to be justified in real time and in the future, a COVID-19 trial response checklist (Table 1) was quickly created in accordance with the best practice available at the time [10]. The checklist systematically addressed the impact and risks relating to recruitment, data quality and completeness, and safe and timely delivery of the trial intervention. Checklists had scope for change should new guidance emerge. Each Trial Management Committee completed the checklist and presented it to the Trial Steering Committee for consideration and endorsement. Once approved, the trial checklist underpinned ancillary documents, including a COVID-19 trial action plan incorporating a communication plan and tracker, to ensure that AKTN’s COVID-19 response and subsequent updates were relayed to stakeholders. The AKTN governance review and decision-making process were standardised for each funded trial (Fig. 2).

**Table 1:** AKTN COVID-19 trial response checklist template

Item/task/stakeholders	Complete
*Communication*	
Initial communication with individual investigators to understand and address local conditions and restrictions	
Strategy for Site Investigators to consult with the Chief Investigator on individual study participant’s safety, welfare and rights are best served by: a) Continuing in the study under the existing protocol, b) Under a modified version, or by c) Discontinuing participation based on the specific circumstances	
Communication plan for regular updates to trial sites	
Resources for site staff to communicate information to participants regarding the research team’s response to COVID-19, and changes to study and monitoring plans that could impact them	
Human Research Ethics Committee notified of participant communication plan	
Communication plan in place for disseminating information to local and global Trial Steering Committee	
Funder communication	
Data and Safety Monitoring Board communication	
*Safety*	
Additional safety monitoring for trial participants who no longer have access to investigational product or the investigational site	
When sites are experiencing pandemic-related employee absenteeism, will reports be stored or submitted later?	
Strategy to record adverse events that have been stored for later reporting	
*Trial conduct*	
Can the trial continue under the existing protocol considering current conditions?^a^ Is an amendment required to the informed consent form?	
Plans in place to delay some assessments for the trial, or stop ongoing recruitment, or withdraw trial participants	
The research team has conducted an evaluation of alternative methods for trial assessments/conduct (phone contacts, virtual visits, home delivery of the investigational product)	
Resources sent to sites to document all COVID-related changes and protocol deviations on a participant-by-participant basis, including the reason for the change or deviation	
Investigational product: determination has been made to either continue the study as per protocol or discontinue the use of an investigational product based on: a) ability to conduct appropriate safety monitoring and/or b) impact on the investigational product supply chain	
*Monitoring*	
Adjust trial monitoring plan to account for COVID-19-related modifications and deviations	
Adjust trial monitoring plan to incorporate increased central and remote monitoring surveillance to maintain site oversight	
*Data management and analysis*	
The research team has considered how the statistical analysis plan and protocol deviations related to COVID-19 will be handled for pre-specified analyses before locking clinical trial database	
A document mapping possible missing data and the information required from sites to explain missing data due to COVID-19 disruptions in the main results publications has been developed^b^	
*Risk management*	
Document the impact of the pandemic on informed consent processes, study visits and procedures, study monitoring and data collection, adverse event reporting, and changes in investigators, site staff and monitors. All policies and procedures will need to continue to be compliant with regional or national policies regarding COVID-19^c^	
Establish and implement trial-specific procedures or revise existing ones to describe how study participants will be protected and how the trial will be managed during possible COVID-19 disruptions	

^a^If emergent or urgent changes are likely to be made to the protocol or informed consent, communicate these to the Human Research Ethics Committee in advance where possible

^b^The expectation is the AKTN will include the relationship of the missing information to COVID-19 and this should be summarised in the final clinical study report

^c^AKTN will need to describe the implemented contingency measures related to COVID-19, including a list of impacted participants by participant number and site (including how the individual’s participation was affected), and provide an analysis and discussion regarding the impact of the contingency actions on safety and efficacy results

### Decision-making

Protecting the wellbeing and safety of participants is a founding principle of GCP and at the forefront of clinical trial design and conduct. Developing a pathway for ethical continuation of trial activities was important: the timely completion of a well-designed and conducted trial has the potential to benefit millions of people. Devising methods to enable the research to continue while under duress acknowledges the valuable contribution of new and already enrolled participants.

Individual trial responses were tailored according to the stage and design of the trial. Initially, the pragmatic design of the majority of AKTN trials, whereby protocols allowed for telehealth or virtual participant visits with exception of the informed consent process and the use of web-based electronic data capture systems for trial data collection, was considered relatively ‘safe’ to facilitate a ‘business as normal’ approach. However, this view was re-evaluated in the context of a rapid surge in the COVID-19 infection rates in the community and the imperative to develop and implement responsive policies and a more pragmatic approach.

### Trial conduct during COVID-19

Recruiting sites were consulted on their capacity to consent participants and collect trial data during the pandemic. Several local Research Governance Offices suspended all new trial-related activity indefinitely, while others limited non-essential research activities, which posed infection control issues, such as patient contact and handling paper consent forms.

Within this framework, site set-up activities, such as execution of clinical trial agreements and collection of GCP essential documents, continued where possible. However, in anticipation of an overwhelmed healthcare system and to avoid unnecessary additional burden to site staff, the Trial Management Committees unilaterally recommended to suspend recruitment of new patients and suspend activation of new sites, a decision supported by the Trial Steering Committees. For patient safety monitoring and preservation of the integrity of the intention to treat analysis of trial data, the Committees also agreed sites should be supported to continue follow-up of already enrolled participants. The pragmatic design of the trials provided the Trial Management Committees with the agility to adjust trial data collection and monitoring processes within the COVID-19 lockdown restrictions, for example highlighting priority trial activities and relaxing the timeframe for data entry (Table 2). Protocol deviations and changes to the trial methodology due to COVID-19, should they occur, were to be reported in the methods section when the trial results manuscript was submitted for publication.

**Table 2:** Modified trial activities

**Trial activities**	**Before COVID-19**	**Revised in response to COVID-19**
***Trial assessments***	Clinic appointments Phone calls	Telehealth appointments Phone calls
***Consent***	Clinic appointments	Telehealth appointments Phone calls and verbal consent Post/email consent forms
***Patient surveys***	Paper survey Email survey Electronic device	Phone calls Post/email survey
***Investigational product***	Hospital pharmacy dispensing	Home delivery
***Training***	On-site initiation meetings Videoconference	Videoconference Videoconference recordings available on demand
***Meetings***	Face to face Videoconference	Videoconference
**Monitoring activities**	**Before COVID-19**	**Revised in response to COVID-19**
***Informed consent process***	On-site visits	Remote monitoring visit phone calls Additional training and resources
***Source data verification***	On-site visits	Remote electronic medical record access Review administrative data sets
***Data quality/trends***	Remote data monitoring	Remote monitoring phone calls Remote data monitoring: - Focus on safety and outcome data - Data entry and query resolution timeframes relaxed during the peak of COVID-19 - Check COVID-19 protocol deviations
***Essential document checks***	On-site visits Electronic Trial Master File platform (limited use)	Written confirmation Electronic Trial Master File platform (*wider use planned*)
***Monitoring checklists***	On-site visit checklist	Remote monitoring visit phone call checklists

Trial conduct decisions were made by the Trial Steering Committees (informed by recommendations from the Trial Management Committees) on behalf of site investigators as part of an intentional strategy to (1) avoid selective site recruitment bias and (2) provide transparency regarding the AKTN’s decision-making process. The screening data were captured during this period to allow a retrospective review of the impact of halting recruitment. The trial activities were revisited at regular intervals by the Trial Management Committees and assessed against the regulatory advice and the best interests of the patients (Table 2).

### Monitoring activities

Prior to COVID-19, all AKTN intervention trials included variable levels of on-site monitoring to check GCP compliance and data quality and integrity, which had to be suspended due to domestic and international travel restrictions.

**Fig. 3:**
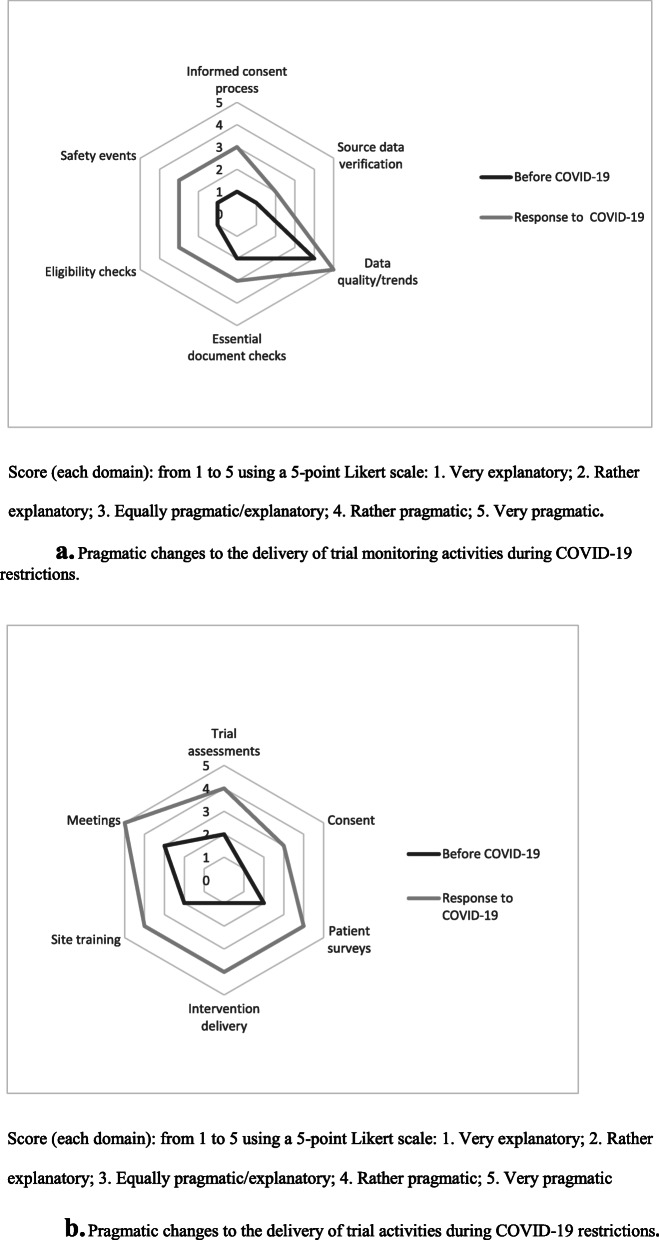
**a** Pragmatic changes to the delivery of trial monitoring activities during COVID-19 restrictions. **b** Pragmatic changes to the delivery of trial activities during COVID-19 restrictions

As a substitute for on-site monitoring, checklists for remote site monitoring visits were created for targeted discussions with site staff about priority data and common queries and risks (Table 2), and more pragmatic monitoring approaches were adopted (Fig. 3a). Prior to conducting remote monitoring calls, sites were given advance notification of which topics would be discussed to facilitate a productive, streamlined discussion. The durations of calls were generally shorter than the time spent with monitors during on-site visits, thereby reducing staff burden.

The AKTN did not favour asking sites to scan redacted documents for remote source data verification as a replacement for on-site monitoring due to labour intensiveness and the risk of compromising patient privacy. Research has shown the majority of significant data queries are not identified through source data verification [12]. Consequently, AKTN has evaluated this as a low-risk activity, and conducting source document verification remotely was not considered a priority. Additionally, the pragmatic conduct of registry-based trials has further reduced the need for source data verification as routine data capture, and existing quality control mechanisms within the registry infrastructure are considered reliable substitutions for source data. Electronic data collection has enabled superior central monitoring through integrated query resolution workflows and enhanced detection of potential data entry errors. Remote monitor access to electronic medical records is permitted by some institutions. This presents a solution to remotely verify trial data veracity and review GCP compliance, particularly consent form completion, patient eligibility and unreported events identification (Table 2 and Fig. 3a).

### Communications

The Human Research Ethics Committees and key stakeholders for each trial were informed in writing of the response plan (Fig. 2). It was important to ensure that a consistent message was conveyed to all site investigators in the climate of heightened uncertainty. Sites were notified of the trial response via email and then called to confirm that they had received the correspondence about the required activities during COVID-19 and to discuss the practicalities of those activities. Investigators were responsible for relaying the appropriate information to participants as well as informing their local research governance to ensure that the measures introduced complied with local advice. Concise website updates, emails, newsletters and electronic data capture system alerts were also used to disseminate the current trial status information. Refresher training on trial site activities was offered for those who had suspended recruitment or opted to implement remote consent. The timings of the next Data and Safety Monitoring Board (DSMB) meetings were flagged for review as the recruitment suspension may have meant that only limited additional data were available for review following the previous meeting.

## Going forward/lessons learned

The decisions to suspend recruitment may have been perceived as premature, considering that the number of the COVID-19 cases that eventuated in Australia and New Zealand to date has been relatively low compared to some other countries. However, at the time of implementing suspension, the COVID-19 infection rate in Australia was doubling every few days at an accelerated rate, and in New Zealand, strict government lockdown measures were implemented to safeguard the nation. The healthcare research workforce activities were diverted away from trials to focus on preparing for the pandemic. At an organisational level, we had to be confident the necessary policies and processes were in place to safeguard all members involved in our network, especially our patients, as well as maintaining trial integrity.

Consumer bodies were not consulted during the pandemic response, except for consumer representatives on Trial Steering Committees. It would be prudent to seek their opinions on the recruitment suspension and changes to the delivery of patient consent and assessments. It remains to be seen if patient uptake into clinical trials will change in light of COVID-19. Patients may be risk averse after such a global crisis, although there has been widespread media coverage about the importance of clinical trials in establishing safe and effective vaccines and treatments for COVID-19, thereby raising awareness of the value of clinical trials and medical research. For those patients who are open to participating but still fearful of attending hospitals, remote consent and assessments should be incorporated into trial protocols to give flexibility and facilitate inclusion. Validation of assessment tools will be required to implement remote assessments, e.g. via telephone. This potential barrier can be addressed via trial embedded research or a “Study Within A Trial (SWAT)” [13].

**Fig. 4:**
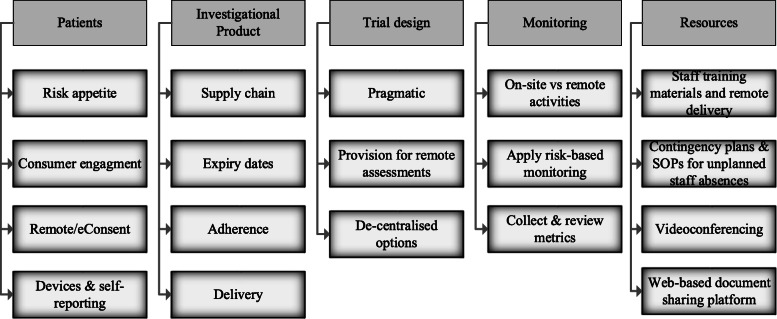
Considerations for the design of future trials

The revised AKTN activities (Table 2) were assessed for their pragmatism across the trial portfolio using a method inspired by the PRECIS-2 tool [14]; there is scope for further pragmatism in trial delivery (Fig. 3b) and monitoring (Fig. 3a). The lessons learned from COVID-19 will mean more weight will be given to innovative and simplified delivery of trial assessments and data acquisition [15] when designing trials to enable trials to continue during future health emergencies (Fig. 4). New trial budgets will include provision for devices, data and additional resources to facilitate remote trial activities and web-based training. Going forward, virtual meetings are likely to be adopted more broadly across site training and monitoring plans. The pandemic has pushed us to explore capabilities to develop rapport via virtual meetings and training sessions, such as redesigning the meeting format to create interactive discussions, polls, question and answers.

In the absence of on-site monitoring visits, where certain activities cannot be done remotely, there is a strong indication for more training, communications (newsletters) and quick reference guides to educate site staff on GCP and prevent errors, rather than react to them. This creates responsible research practice, empowers the research team and instils confidence as we move away from traditional on-site monitoring. The acceptability of the phone calls for both the monitor and site staff is currently under review, and the processes (Table 2) will be adapted according to feedback from all stakeholders. Documented decisions, evaluation of processes, and routine tracking of metrics will enable an impact assessment [16] and inform the design of future trial protocols and monitoring plans (Fig. 4).

This paper has not addressed statistical issues. The potential effect of the COVID-19 pandemic on power calculations was considered at the start of the pandemic, and any new developments potentially altering sample size considerations are being monitored. In accordance with recommendations [17, 18], statistical analysis plans will be reviewed and modified where necessary to accommodate any impact of COVID-19 on interventions and trial data and additional sensitivity and supplemental analyses will be added to the analysis plans with the aim of fully understanding the effect of COVID-19 on trial results and interpretation.

## Conclusion

The clinical trial network was not prepared to conduct trials safely during a global pandemic. In order to ensure trial integrity while safeguarding outcome for all stakeholders involved in clinical trials, the decision was made to suspend recruitment, re-evaluate and strategically develop policies to facilitate conduct of high-quality clinical trials adapted to different approaches necessitated in the context of COVID-19. It is too early to assess the long-term impact of COVID-19 on existing trials, but its occurrence has reshaped the organisation and processes to better prepare for and have the agility to adapt to future disruptions caused by the re-emergence of the virus.

## Data Availability

Not applicable.

## References

[1] Hsu CM, Weiner DE (2020). COVID-19 in dialysis patients: outlasting and outsmarting a pandemic. Kidney Int.

[2] Jager KJ, Kramer A, Chesnaye NC, Couchoud C, Sánchez-Álvarez JE, Garneata L, Collart F, Hemmelder MH, Ambühl P, Kerschbaum J, Legeai C, del Pino y Pino MD, Mircescu G, Mazzoleni L, Hoekstra T, Winzeler R, Mayer G, Stel VS, Wanner C, Zoccali C, Massy ZA (2020). Results from the ERA-EDTA Registry indicate a high mortality due to COVID-19 in dialysis patients and kidney transplant recipients across Europe. Kidney Int.

[3] Caillard S, Anglicheau D, Matignon M, Durrbach A, Greze C, Frimat L, Thaunat O, Legris T, Moal V, Westeel PF, Kamar N, Gatault P, Snanoudj R, Sicard A, Bertrand D, Colosio C, Couzi L, Chemouny JM, Masset C, Blancho G, Bamoulid J, Duveau A, Bouvier N, Chavarot N, Grimbert P, Moulin B, le Meur Y, Hazzan M, Caillard S, Moulin B, Fafi-Kremer S, Hazzan M, Anglicheau D, Hertig A, Tourret J, Barrou B, Morelon E, Thaunat O, Couzi L, Merville P, Moal V, Legris T, Westeel PF, Jaureguy M, Frimat L, Ducloux D, Bamoulid J, Bertrand D, Tsimaratos M, Garaix-Gilardo F, Dumortier J, Mussot S, Roux A, Sebbag L, le Meur Y, Blancho G, Masset C, Kamar N, Francois H, Rondeau E, Bouvier N, Mousson C, Buchler M, Gatault P, Augusto JF, Duveau A, Vigneau C, Morin MC, Chemouny J, Golbin L, Grimbert P, Matignon M, Durrbach A, Greze C, Snanoudj R, Colosio C, Schvartz B, Malvezzi P, Mariat C, Thierry A, le Quintrec M, Sicard A, Rerolle JP, Heng AÉ, Garrouste C, Coponat HV, Epailly É, Brugiere O, Dharancy S, Salame É, Saliba F (2020). An initial report from the French SOT COVID Registry suggests high mortality due to Covid-19 in recipients of kidney transplants. Kidney Int.

[4] Morrish AT, Hawley CM, Johnson DW, Badve SV, Perkovic V, Reidlinger DM, Cass A (2014). Establishing a clinical trials network in nephrology: experience of the Australasian Kidney Trials Network. Kidney Int.

[5] Tong A, Crowe S, Chando S, Cass A, Chadban SJ, Chapman JR, Gallagher M, Hawley CM, Hill S, Howard K, Johnson DW, Kerr PG, McKenzie A, Parker D, Perkovic V, Polkinghorne KR, Pollock C, Strippoli GFM, Tugwell P, Walker RG, Webster AC, Wong G, Craig JC (2015). Research priorities in CKD: report of a national workshop conducted in Australia. Am J Kidney Dis.

[6] Dansie K, Viecelli AK, Pascoe EM, Johnson DW, McDonald S, Clayton P, et al. Novel trial strategies to enhance the relevance, efficiency, effectiveness and impact of nephrology research. Kidney Int. 2020;98(3):572–8. 10.1016/j.kint.2020.04.050. 10.1016/j.kint.2020.04.050 32464216

[7] Ford I, Norrie J (2016). Pragmatic trials. N Engl J Med.

[8] Transcelerate. The TransCelerate model approach [Available from: https://transceleratebiopharmainc.com/rbminteractiveguide/how-does-clinical-trial-site-monitoring-work-under-a-risk-based-monitoring-approach/the-transcelerate-model/. Accessed 25 May 2020.

[9] Australian Clinical Trials Alliance. COVID-19: guidance on Australian clinical trials for institutions, HRECs, researchers and sponsors 2020 [Available from: https://clinicaltrialsalliance.org.au/latest-news/25-03-resources-on-covid-19-for-the-clinical-trial-sector/. Accessed 25 May 2020.

[10] Food and Drug Administration. FDA guidance on conduct of clinical trials of medical products during COVID-19 public health emergency. Guidance for Industry, Investigators, and Institutional Review Boards 2020 [Available from: https://www.fda.gov/media/136238/download. Accessed 25 May 2020.

[11] Group CTPR. COVID-19: guidance on clinical trials for institutions, HRECs, researchers and sponsors 2020 [Available from: https://www.nhmrc.gov.au/sites/default/files/documents/attachments/ctprg-statement-clinical-trials-covid.pdf. Accessed 25 May 2020.

[12] Andersen JR, Byrjalsen I, Bihlet A, Kalakou F, Hoeck HC, Hansen G, Hansen HB, Karsdal MA, Riis BJ (2015). Impact of source data verification on data quality in clinical trials: an empirical post hoc analysis of three phase 3 randomized clinical trials. Br J Clin Pharmacol.

[13] Treweek S, Bevan S, Bower P, Campbell M, Christie J, Clarke M, Collett C, Cotton S, Devane D, el Feky A, Flemyng E, Galvin S, Gardner H, Gillies K, Jansen J, Littleford R, Parker A, Ramsay C, Restrup L, Sullivan F, Torgerson D, Tremain L, Westmore M, Williamson PR (2018). Trial forge guidance 1: what is a study within a trial (SWAT)?. Trials..

[14] Loudon K, Treweek S, Sullivan F, Donnan P, Thorpe KE, Zwarenstein M (2015). The PRECIS-2 tool: designing trials that are fit for purpose. BMJ.

[15] Inan OT, Tenaerts P, Prindiville SA, Reynolds HR, Dizon DS, Cooper-Arnold K, Turakhia M, Pletcher MJ, Preston KL, Krumholz HM, Marlin BM, Mandl KD, Klasnja P, Spring B, Iturriaga E, Campo R, Desvigne-Nickens P, Rosenberg Y, Steinhubl SR, Califf RM (2020). Digitizing clinical trials. NPJ Digit Med.

[16] Constable L, Davidson T, Breeman S, Cotton S, McDonald A, Wileman S, Norrie J (2020). How to deal with a temporary suspension and restarting your trial: our experiences and lessons learnt. Trials..

[17] Meyer RD, Ratitch B, Wolbers M, Marchenko O, Quan H, Li D, Fletcher C, Li X, Wright D, Shentu Y, Englert S, Shen W, Dey J, Liu T, Zhou M, Bohidar N, Zhao PL, Hale M (2020). Statistical issues and recommendations for clinical trials conducted during the COVID-19 pandemic. Stat Biopharm Res.

[18] Cro S, Morris TP, Kahan BC, Cornelius VR, Carpenter JR (2020). A four-step strategy for handling missing outcome data in randomised trials affected by a pandemic. BMC Med Res Methodol.

